# Fraxini cortex (Qinpi): reframing a traditional heat-clearing botanical drug as a systemic immune-metabolic regulator

**DOI:** 10.3389/fphar.2026.1799355

**Published:** 2026-06-17

**Authors:** Ping Xin, Yi-Hua Fan, Jing-Yan Xu, Shi-jie Mao, Ji-Wei Zhang, Zhi-li Luo, Xia Zhou

**Affiliations:** 1 School of Traditional Chinese Medicine, Tianjin University of Traditional Chinese Medicine, Jinghai, Tianjin, China; 2 Department of Rheumatism and Immunity, Hospital of Chengdu University of Traditional Chinese Medicine, Chengdu, Sichuan, China; 3 School of Clinical Medicine, Chengdu University of Traditional Chinese Medicine, Chengdu, Sichuan, China; 4 Haihe Laboratory of Modern Chinese Medicine, Tianjin, China; 5 Department of Pharmacy, Quzhou People’s Hospital (The Affiliated Quzhou Hospital of Wenzhou Medical University), Quzhou, Zhejiang, China; 6 Tuina and Rehabilitation Ward, Yibin Hospital of integrated traditional Chinese and Western Medicine, Yibin, Sichuan, China; 7 Department of Integrated Traditional Chinese and Western Medicine, The First People’s Hospital of Zigong, Zigong, Sichuan, China

**Keywords:** anti-inflammatory, esculin, Fraxini Cortex, immunomodulation, modernization of traditional Chinese medicine, multi-target therapy, pharmacological mechanisms, phytochemistry

## Abstract

Fraxini Cortex (Qinpi), a traditional Chinese medicine used for millennia for “Qingre” (heat-clearing), is now being re-evaluated in the context of modern immunology and systems pharmacology. Its traditional applications in treating inflammatory conditions hint at a broader, systemic regulatory capacity. This review proposes that Fraxini Cortex functions as a systemic immune-metabolic regulator. We aim to synthesize existing evidence through this novel lens, hypothesizing that its therapeutic potential stems from the synergistic ability of its constituents to target pivotal hubs at the interface of immune response and cellular metabolism. We conducted a comprehensive literature review encompassing the phytochemistry, pharmacology, pharmacokinetics, and clinical evidence of Fraxini Cortex. Phytochemically, Fraxini Cortex is rich in coumarins (e.g., esculetin, esculin), secoiridoids, and phenylethanoid glycosides. Pharmacologically, its mechanisms extend beyond mere anti-inflammation. Crucially, we propose that its constituents reprogram tumor metabolism by targeting the glycolytic enzyme GPI (e.g., oleuropein); execute an anti-virulence strategy against MRSA by inhibiting Sortase A (e.g., esculetin); and correct uric acid and glucose-lipid metabolic disorders by modulating urate transporters (URAT1/GLUT9/ABCG2) and insulin signaling (e.g., fraxin and esculin). This hypothesis is based on the convergence of evidence showing that these multi-target actions are integrated by its capacity to suppress core inflammatory pathways (NF-κB, MAPK) while activating antioxidant defenses (Nrf2), thereby promoting the resolution of inflammation and tissue homeostasis. Pharmacokinetic challenges, notably low oral bioavailability of key coumarins, are being addressed by novel drug delivery systems. Preliminary clinical studies support its benefits in psoriasis and gouty arthritis. In conclusion, we propose that Fraxini Cortex exemplifies how a traditional “heat-clearing” botanical drug can be reinterpreted as a multi-target regulator of immune-metabolic homeostasis. Future research should focus on validating this paradigm using omics technologies, developing bioavailability-enhanced formulations, and conducting rigorous clinical trials for immune-metabolic diseases. This shift in perspective may accelerate the translation of Fraxini Cortex and similar botanical drugs into standardized, evidence-based immunomodulatory therapies.

## 1 Introduction

Fraxini Cortex, derived from the dried branch bark or stem bark of Fraxinus rhynchophylla Hance, F. chinensis Roxb., F. szaboana Lingelsh., or F. stylosa Lingelsh. (Family Oleaceae) (Chinese Pharmacopoeia Commission, 2020), holds a distinguished reputation in Traditional Chinese Medicine (TCM). Its medicinal use dates back to the Eastern Han Dynasty, documented in the ancient classic “Shennong Bencao Jing” (circa 200–250 AD), where it was classified as a “superior-grade” medicinal substance. Within the TCM theoretical framework, its properties are described as bitter, astringent, and cold, with attributed meridians to the liver, gallbladder, and large intestine. This profile underpins its traditional applications in clearing heat, drying dampness, checking diarrhea, and improving vision by clearing corneal opacity (Zhao et al., 2024). Consequently, it has been a principal botanical drug for treating conditions like dysentery characterized by dampness-heat and eye disorders such as conjunctival congestion and pain, featuring prominently in famous formulas like Baitouweng Decoction (Qiu et al., 2022; Wang F.Y. et al., 2020; Wang et al., 2019).

However, the understanding of Fraxini Cortex is undergoing a significant paradigm shift. The traditional TCM concept of “clearing heat” is now broadly interpreted in modern biomedical terms to encompass a wide spectrum of activities, including broad-spectrum anti-inflammatory, antioxidant, and immunomodulatory effects (Hu et al., 2025). Inflammation, as a universal pathological process, serves as the common thread linking its historical uses with emerging pharmacological roles. Thus, propelled by advances in modern separation techniques, analytical technologies, and molecular biology, Fraxini Cortex is transitioning from an empirically used traditional remedy to a rich source of bioactive compounds and a unique window for exploring multi-target pharmacology in complex diseases (Yang et al., 2016; Nie et al., 2016; Zheng X. et al., 2024).

Systematic phytochemical investigations have identified over 132 chemical constituents from Fraxini Cortex. Among these, coumarins, particularly esculetin (also known as aesculetin or Fraxetin) and its derivatives, are recognized as the primary pharmacologically active basis (Wang et al., 2016a; Yan et al., 2023; Liang et al., 2017). More intriguingly, its multi-target, integrative regulatory mechanisms are being progressively deciphered. Both esculin and esculetin have been demonstrated to possess significant pharmacological activities, including anti-inflammatory, antioxidant, anti-diabetic, anti-cancer, antimicrobial, antiviral, and neuroprotective effects (Li C. X. et al., 2022; Zhao and Yan, 2022). Inhibition of pivotal inflammatory signaling hubs such as NF-κB and MAPK pathways (Ni et al., 2023; Cai and Cai, 2023; Yao et al., 2022; Li et al., 2020a; Li et al., 2019) provides a molecular foundation for its core “heat-clearing” effect. This foundational anti-inflammatory activity extends to innovative therapeutic strategies. For instance, its anti-inflammatory action manifests an “anti-virulence strategy,” reducing tissue damage by suppressing excessive host inflammatory responses triggered by bacterial virulence factors (Li et al., 2020a; Li et al., 2019). It also shows promise in directly targeting cancer metabolism (Cai et al., 2024; Feng et al., 2025)—processes profoundly influenced by the inflammatory microenvironment. Furthermore, its emerging potential in managing metabolic disorders (e.g., hyperuricemia) (Huang et al., 2023; Zhou et al., 2018; Wang et al., 2016b; Liu and Liu, 2020), neurological diseases (Yang et al., 2025; Yao et al., 2022), and osteoporosis (Zheng Z. et al., 2024) is increasingly understood to be mediated through mechanisms like ferroptosis inhibition, mitigation of chronic/low-grade inflammation, and alleviation of oxidative stress.

However, despite these advances, a unifying conceptual framework that transcends a mere listing of its multi-target effects is lacking. Most studies have cataloged its anti-inflammatory, antioxidant, and metabolic properties in isolation, without interrogating how these diverse actions are interconnected to produce a systemic therapeutic outcome. This gap limits our ability to fully exploit its potential and design rational clinical applications.

### 1.1 Literature search strategy

To ensure comprehensiveness, we systematically searched PubMed, Web of Science, China National Knowledge Infrastructure (CNKI), and Wanfang Data databases from their inception to December 2025. Search terms mainly included: “fraxini cortex,” “Qinpi,” “esculin,” “esculetin,” “fraxin,” “fraxetin,” “anti-inflammatory,” “immunomodulation,” “metabolism,” “pharmacokinetics,” and corresponding Chinese keywords. Inclusion criteria were original research articles and reviews related to the phytochemistry, pharmacological mechanisms, pharmacokinetics, and clinical applications of Fraxini Cortex. We excluded non-English/Chinese publications, conference abstracts, and articles for which full text was unavailable (See the Supplementary Material).

### 1.2 Aims and hypothesis

This review aims to provide a critical and updated synthesis of Fraxini Cortex research by proposing and employing a novel perspective: Fraxini Cortex as a systemic immune-metabolic regulator. We define a “systemic immune-metabolic regulator” as a multi-target agent capable of simultaneously modulating dysfunctional immune responses and the underlying metabolic dysregulation that fuels them, thereby restoring homeostasis through action on key nodes where immune signaling and metabolic pathways intersect (e.g., metabolic enzymes GPI, inflammasome NLRP3, antioxidant hub Nrf2). Through this lens, we will re-interpret its phytochemistry as a source of multi-target ligands for immune-metabolic hubs. We will systematically reconstruct its pharmacological network, highlighting how actions like GPI inhibition, SrtA antagonism, and urate transporter regulation converge on immune-metabolic crosstalk. We will critically evaluate its pharmacokinetic profile and the emerging clinical evidence. Finally, we will outline a forward-looking research agenda derived from this framework, aiming to bridge traditional wisdom with contemporary precision medicine in the fight against chronic immune-metabolic diseases.

## 2 Phytochemical basis of fraxini cortex

### 2.1 Major active constituents of fraxini cortex

The pharmacological actions of Fraxini Cortex are rooted in its complex and synergistic chemical composition (Table 1). Through modern chromatographic and spectroscopic techniques, such as UPLC-Q-Exactive Orbitrap MS and GC-MS, researchers have systematically characterized its chemical constituents (Wang et al., 2016a; Yan et al., 2023). Pharmacognostic studies provide a basis for the authentication and quality control of Fraxini Cortex, aiding in its distinction from common adulterants (Tian et al., 2017). Its major constituents can be systematically categorized as follows (Liu et al., 2016), collectively forming the material foundation for its multi-target pharmacological effects.

**TABLE 1:** Main active metabolites of cortex fraxini and their structural characteristics.

Metabolite class	Representative constituents	Molecular formula	Molecular weight	Chemical structure	REF
Coumarins	Esculin	C_15_H_16_O_9_	340.28 g/mol	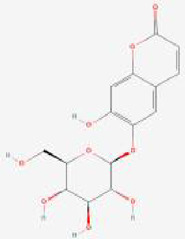	Li C. X. et al. (2022)
	Esculetin	C_9_H_6_O_4_	178.14 g/mol	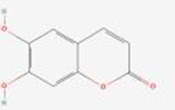	Li et al. (2022)
	Fraxetin	C_10_H_8_O_5_	208.16 g/mol	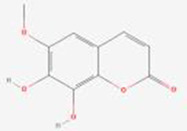	Cai et al. (2024)
	Fraxin	C_16_H_18_O_10_	370.31 g/mol	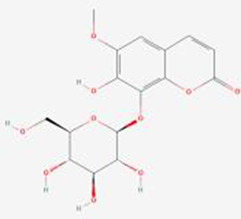	Huang et al. (2023)
	Scopoletin	C_10_H_8_O_4_	192.17 g/mol	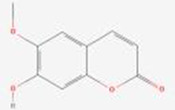	Wu et al. (2019)
	Scopolin	C_16_H_18_O_9_	354.31 g/mol	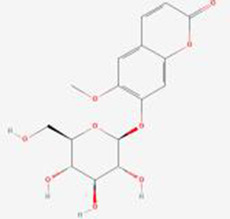	Yan et al. (2023)
	Isofraxidin	C_11_H_10_O_5_	222.19 g/mol	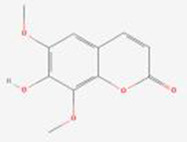	Yan et al. (2023)
Secoiridiod glycosides	Oleuropein	C_25_H_32_O_13_	540.51 g/mol	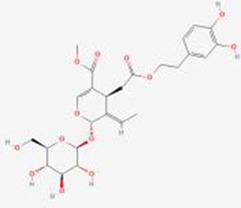	Wang et al. (2016a)
	Ligustroside	C_25_H_32_O_13_	540.51 g/mol	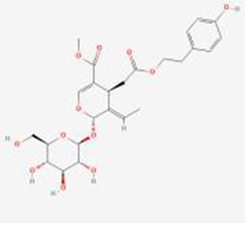	Wang et al. (2016a)
Phenylethanoid glycosides	Salidroside	C_14_H_20_O_7_	300.30 g/mol	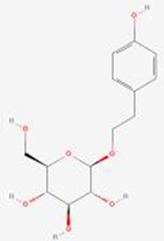	Wang et al. (2016a)
Lignans and flavonoids	Pinoresinol	C_20_H_22_O_6_	358.38 g/mol	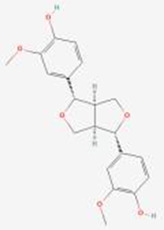	Wang et al. (2016a)

Firstly, coumarins represent the most characteristic and bioactive core metabolites of Fraxini Cortex, serving as the reference standards for quality control in the Chinese Pharmacopoeia (Pan et al., 2016; Zhang et al., 2019; Fu, 2014). Esculin (Aesculin) and its aglycone esculetin (Aesculetin) are the most extensively studied pair of compounds in Fraxini Cortex (Chu and Cai, 2024; Qian et al., 2023; Wang et al., 2018). As a glycoside, esculin possesses relatively good water solubility; it is readily hydrolyzed *in vivo* by gut microbiota or enzymes to its aglycone esculetin, which is considered the key direct active molecule responsible for various pharmacological effects such as anti-inflammatory and anti-tumor activities (Liao and Chen, 2025). The 6,7-catechol hydroxyl structure of esculetin is a crucial pharmacophore for its antioxidant, anti-tumor properties and interaction with specific targets (e.g., GPI) (Hong, 2022; Wu, 2020; Zhang, 2016). Fraxin and its aglycone fraxetin constitute another important pair of coumarins (Ha and Son, 2024). The presence of an 8-methoxy and a 7-hydroxy group in fraxetin’s structure confers a unique activity profile (Tang et al., 2024), demonstrating significant effects in areas such as antioxidant activity (Sun et al., 2024; Luo et al., 2023), antibacterial action, and promotion of bone healing (Zheng Z. et al., 2024). Other coumarins, including scopolin, scopoletin, and isofraxidin, contribute to the diversity and functional complementarity of the coumarin family in Fraxini Cortex (Wang et al., 2016a; Yan et al., 2023; Liu et al., 2016).

Secoiridoids, such as oleuropein and ligstroside, are renowned for their potent antioxidant and anti-inflammatory activities, working synergistically with coumarins to enhance the overall therapeutic efficacy of Fraxini Cortex (Yang et al., 2016). Studies indicate that oleuropein itself exhibits significant hepatoprotective effects and anti-tumor potential, potentially acting through independent or intersecting pathways with coumarins (Hong, 2022). Phenylethanoid glycosides, like salidroside and hydroxytyrosol glucoside, not only contribute to antioxidant activity but also show promise in neuroprotection and anti-fatigue effects (Wang et al., 2016a; Wang Y. et al., 2022). The presence of salidroside provides a chemical basis for the potential adaptogen-like and neuroprotective effects of Fraxini Cortex (Yang et al., 2025). Lignans (e.g., pinoresinol, syringaresinol) and flavonoids (e.g., quercetin, kaempferol) further broaden the spectrum of anti-inflammatory, antioxidant, and cytoprotective actions of Fraxini Cortex (Wang et al., 2016a; Yan et al., 2023; Yang et al., 2016). Although present in relatively lower concentrations, these constituents may play important auxiliary and synergistic roles within the holistic “sovereign-minister-assistant-courier” framework of the botanical drug.

### 2.2 Analytical methods and quality control of chemical constituents

A comprehensive system integrating classical and modern techniques has been established for the systematic analysis and quality control of Fraxini Cortex’s chemical constituents. For chemical analysis, High-Performance Liquid Chromatography (HPLC) and Ultra-Performance Liquid Chromatography (UPLC) coupled with Ultraviolet (UV) or Mass Spectrometry (MS) detectors are the core technologies for qualitative and quantitative analysis (Li Z. Y. et al., 2022; Qian et al., 2023; Pan et al., 2016; Li et al., 2016d; Chen et al., 2017). The application of high-resolution mass spectrometry techniques, such as UPLC-Q-Exactive Orbitrap MS, has proven to be a powerful tool for elucidating the complex chemical profile of Fraxini Cortex (Wang et al., 2016a; Yan et al., 2023). By optimizing chromatographic conditions (e.g., using a Halo 90Å C18 column with a gradient elution of 0.05% formic acid in water-acetonitrile) and combining electrospray ionization in both positive and negative ion modes, comprehensive profiling has been achieved. These studies have successfully identified 72 chemical constituents, including 20 coumarins, 8 secoiridoids, 10 phenylethanoid glycosides, 17 flavonoids, 16 organic acids, and 1 other metabolite, with 36 being reported for the first time from this botanical drug (Wang et al., 2016a; Yan et al., 2023). For rapid identification, Direct Analysis in Real Time Mass Spectrometry (DART-MS) has also been successfully applied.

For quality control, a standardized methodological system has been established. The methods included in the Chinese Pharmacopoeia encompass macroscopic identification, microscopic identification, Thin-Layer Chromatography (TLC) identification, and assay determination. Macroscopic identification focuses on observing its rolled or trough-shaped branch bark, specific color and texture of the outer and inner surfaces, fracture characteristics, and bitter taste. Microscopic identification reveals features such as the cork layer, stone cells and fiber bundles in the cortex and pericycle, a crisscross pattern of fiber bundles and stone cells, and cells containing calcium oxalate sand crystals. TLC identification often uses esculin, esculetin, and fraxetin as reference standards, employing solvent systems like chloroform-methanol-formic acid or the more environmentally friendly dichloromethane-methanol-formic acid, with improved methods yielding clear spots, good resolution, and high reproducibility (Jiang et al., 2015). The assay determination relies primarily on HPLC (Li et al., 2016d), typically using an octadecylsilyl silica gel column, acetonitrile-0.1% phosphoric acid solution as the mobile phase, and detection at 334 nm to determine the total content of esculin and esculetin (required to be not less than 1.0%).

Furthermore, various modern analytical techniques have expanded the arsenal for quality control. Fingerprinting technology combined with chemometrics (e.g., Principal metabolite Analysis, Cluster Analysis) is widely used to comprehensively evaluate the consistency of botanical drug quality and can effectively differentiate samples from different botanical origins (e.g., F. rhynchophylla, F. szaboana), geographical locations, and plant parts (branch bark vs. stem bark) (Pan et al., 2016; Zhang et al., 2019; Shi et al., 2020; Zhou, 2021; Cao et al., 2023). The establishment of the Quantitative Analysis of Multi-metabolites by Single Marker (QAMS) method allows for the simultaneous determination of multiple coumarin metabolites using a single reference standard, improving analytical efficiency and reducing costs (Cao et al., 2023; Xue, 2021). Highly sensitive analytical techniques, such as an electrochemical sensor based on a TiO_2_-PDDA-Gr nanocomposite, have achieved a detection limit for esculetin as low as 4 × 10^−9^ mol/L (Li et al., 2016a). A cloud point extraction-HPLC method using Genapol X-080 as the extraction agent yielded recoveries of 95.3% and 96.0% for esculin and esculetin, respectively, with RSDs less than 2.3% (Wang and Yang, 2015). Non-aqueous Capillary Electrophoresis allows for the simultaneous determination of fraxin, esculin, and esculetin with detection limits ranging from 0.1557 to 0.5382 μg/mL (Li et al., 2005). Polarography also provides a viable option for the determination of esculetin (Zhang and Xu, 1982).

Research on optimizing the extraction process for Fraxini Cortex provides assurance for quality controllability. Optimization methods like orthogonal tests have determined the optimal extraction parameters for formulations containing Fraxini Cortex, such as the Xikebao prescription (Leng et al., 2016). Ultrasound-assisted extraction of Fraxini Cortex coumarins, optimized by response surface methodology, achieved a maximum yield of 7.136% ± 0.254% under optimal conditions: temperature 61.92 °C, ethanol concentration 59.94%, liquid-to-solid ratio 9.96:1, and extraction time 40.79 min (Jia and Zhang, 2025). Surfactant-assisted extraction yielded up to 1.038% coumarins under specific conditions (Tong et al., 2021). Comparative studies show that alcohol extraction (refluxing twice with 8-fold 75% ethanol for 2 h each) significantly outperforms water extraction in terms of extract yield, total coumarin content, and the total content of four specific coumarins, achieving a total coumarin extraction rate of 6.62% (Wang et al., 2015a). Optimization of ultrasound-assisted extraction of Fraxini Cortex polyphenols by response surface methodology identified optimal parameters: 45% ethanol, 33 min ultrasonic time, 90 W power, and a 1:26 solid-to-liquid ratio. The extract exhibited IC_50_ values of 6.217 and 4.048 μg mL^−1^ against DPPH and ABTS radicals, respectively, demonstrating antioxidant activity comparable to vitamin C (Liu Y. H. et al., 2019).

In the quality control of compound preparations, analytical methods for Fraxini Cortex are also widely applied. For example, for the Erihemu-8 powder, an HPLC fingerprint was established with 17 common peaks (similarity >0.9) alongside the determination of esculetin content. For Bawet Qinpi pills, microscopic identification, TLC, and HPLC are comprehensively used for the qualitative identification and content determination of esculin, esculetin, and 7-hydroxycoumarin. For Compound Baitouweng granules, Fraxini Cortex is identified by TLC, and the content of Scutellaria baicalensis is determined by HPLC (Ma and Tang, 2018). In quality standard studies for preparations like Fraxini Cortex dispensing granules and Xikebao capsules, TLC and HPLC are extensively used for the qualitative identification of Fraxini Cortex and the quantitative determination of marker metabolites like esculin and esculetin, ensuring stable and controllable quality of the formulations (Guo et al., 2016; Pan et al., 2016). Together, these rigorous and diverse analytical methods form a solid foundation ensuring the stable quality of Fraxini Cortex crude drug and its preparations, thereby supporting the reliability and reproducibility of pharmacological research and clinical application outcomes.

## 3 Pharmacological effects and molecular mechanisms

**FIGURE 1:**
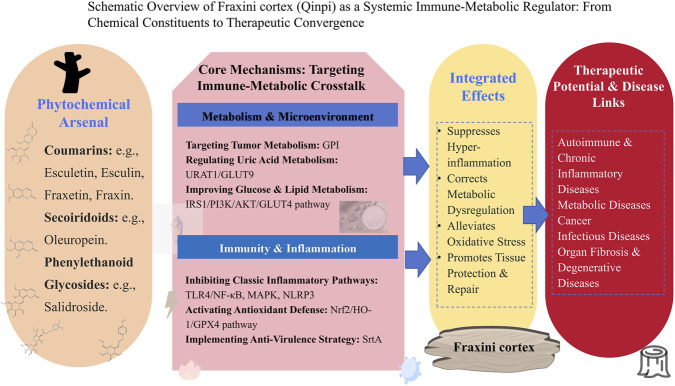
Schematic overview of fraxini cortex (Qinpi) as a systemic immune-metabolic regulator: From chemical constituents to therapeutic convergence.

The diverse therapeutic effects of Qinpi, traditionally categorized under “heat-clearing,” can be coherently understood through the framework of immune-metabolic regulation. Its active metabolites do not merely suppress inflammation in isolation; instead, they target key nodes where immune signaling and metabolic pathways intersect, leading to a more integrated restoration of tissue function. Importantly, we distinguish throughout between effects demonstrated with isolated compounds (e.g., esculin, esculetin, fraxin) and those shown with whole herbal extracts. Where not specified, findings typically derive from studies on purified compounds. The following sections detail these mechanisms, organized to highlight their contribution to this overarching theme (Table 2; Figure 1).

**TABLE 2:** Pharmacological effects of fraxini cortex (Qinpi) and its active metabolites.

Core active metabolite	Study model/Disease	Evidence type	Primary pharmacological effect	Mechanism of action	Key signaling pathways/Targets	Ref
Esculin	Anti - inflammatory					
	Ulcerative colitis	in vitro, *in vivo*	Anti-inflammatory	Activates PPARγ and inhibits the NF-κB pathway, reducing pro-inflammatory cytokine production	PPARγ, NF-κB	Tian et al. (2019)
	Septic cardiomyopathy	in vitro, *in vivo*	Cardioprotective, anti-inflammatory	Directly binds to TLR4, inhibiting LPS-induced TLR4 upregulation and subsequent NF-κB p65 phosphorylation	TLR4, NF-κB	Su et al. (2024)
	Acute lung injury (ALI)	in vitro, *in vivo*	Anti - inflammatory, inhibits neutrophil migration	Directly interacts with β2 integrin on neutrophils, reducing affinity for ICAM - 1 and disrupting cytoskeletal remodeling via the Vav1/Rac1/PAK1/LIMK1/cofilin axis	β2 integrin, ICAM - 1	Ni et al. (2023)
	Endotoxic shock	in vitro, *in vivo*	Anti - inflammatory	Inhibits NF - κB activation, reducing NO and pro - inflammatory cytokine production	NF - κB	Li et al. (2016c)
	Myocardial ischemia - reperfusion injury (MIRI)	in vitro, *in vivo*	Cardioprotective, inhibits pyroptosis	Activates the Akt/GSK3β pathway, thereby inhibiting NF - κB activation and NLRP3 inflammasome - mediated pyroptosis	Akt/GSK3β, NF - κB, NLRP3	Xu et al. (2021)
Esculin	Anti - tumor					
	Colorectal cancer (CRC)	in vitro, *in vivo*	Induces apoptosis and ferroptosis	Triggers ER stress, inducing apoptosis via the PERK/eIF2α/CHOP pathway and ferroptosis via the Nrf2/HO - 1 axis	PERK/eIF2α/CHOP, Nrf2/HO - 1	Ji et al. (2024)
Esculin	Metabolic and cardiovascular regulation					
	Obesity - induced insulin resistance	in vivo	Improves insulin sensitivity	Remodels adipose tissue and directly activates the IRS1/PI3K/AKT/GLUT4 pathway in adipocytes to enhance glucose uptake	IRS1/PI3K/AKT, GLUT4	Yang et al. (2024)
	Atherosclerosis	in vivo	Anti - atherosclerotic, improves lipid profile	Inhibits the DPP4/NF - κB signaling pathway, ameliorating dyslipidemia and inflammation	DPP4, NF - κB	Zhang L. L. et al. (2025)
Esculin	Organ and tissue protection					
	Non - alcoholic fatty liver disease (NAFLD)	in vitro	Hepatoprotective, reduces steatosis	Suppresses the PERK/eIF2A/ATF4 ER stress pathway, alleviating hepatocyte lipid accumulation	PERK/eIF2A/ATF4	Xu et al. (2024)
	Alzheimer’s disease (*C. elegans* model)	in vivo (*C. elegans*)	Neuroprotective	Activates stress regulators DAF - 16 and HSF - 1, enhancing antioxidant defense against Aβ toxicity	DAF - 16, HSF - 1	Wang Q. H. et al. (2022)
Esculetin	Anti - tumor					
	Hepatocellular carcinoma (HCC)	in vitro, *in vivo*	Induces ferroptosis	Inhibits the Nrf2 - xCT/GPX4 axis, leading to Fe^2+^ accumulation, GSH depletion, and lipid peroxidation	Nrf2 - xCT/GPX4	Qu et al. (2025)
	Triple - negative breast cancer (TNBC)	in vitro	Induces apoptosis	Inhibits the JAK2/STAT3 pathway, activating the caspase cascade (caspase - 9, - 3, PARP cleavage)	JAK2/STAT3, caspase	Feng et al. (2025)
Esculetin	Anti - inflammatory and antioxidant					
	Sepsis - induced lung inflammation	in vitro, *in vivo*	Anti - inflammatory	Inhibits NF - κB and STAT1/STAT3 signaling pathways in macrophages	NF - κB, STAT1/STAT3	Cheng et al. (2021)
	Allergic asthma	in vivo	Immunomodulatory	Corrects Th1/Th2 imbalance, reducing IL - 4 and IgE while increasing IFN - γ	Th1/Th2 cytokines	Li et al. (2020b)
	Oxidative stress (review)	in vitro, *in vivo*	Potent antioxidant	Activates the Nrf2 pathway and directly scavenges free radicals (catechol structure is key)	Nrf2	Cai and Cai, 2023; Luo et al., 2023; Yang et al., 2025; Ju et al., 2024
Esculetin	Organ and tissue protection					
	Intracerebral hemorrhage (ICH)	in vitro, *in vivo*	Neuroprotective, inhibits ferroptosis	Promotes NUDT1 - mediated m^7^G methylation of GPX4 mRNA, enhancing its stability and inhibiting neuronal ferroptosis	NUDT1, GPX4	Gong et al. (2025)
	Diabetic Retinopathy	in vitro	Protects retinal cells	Activates the HIF - 1α/BNIP3 pathway to induce protective autophagy under high glucose stress	HIF - 1α/BNIP3	Chen et al. (2022)
	Osteoporosis	in vivo	Anti - osteoporotic	Downregulates serum IL - 6 and the RANKL/OPG pathway, suppressing osteoclast activity	IL - 6, RANKL/OPG	Liu et al., 2018, Liu Y. H. et al., 2019
	Atherosclerosis (review)	in vitro, *in vivo*	Anti - atherosclerotic	Multi - mechanistic: Lowers lipids, inhibits VSMC proliferation, prevents LDL oxidation, and exerts anti - inflammatory effects	Multiple	Wang Y. et al. (2022b)
Fraxin	Anti-inflammatory and immunomodulation					
	Ulcerative colitis	in vitro, *in vivo*	Anti-inflammatory	Modulates oxidative stress and inflammation via TLR4/NF-κB and MAPK pathways	TLR4/NF-κB, MAPK	Sun et al. (2024)
	Acute lung Injury/Acute respiratory distress syndrome (ALI/ARDS)	in vitro, *in vivo*	Anti-inflammatory, protective	Downregulates NF-κB and MAPK pathways, reducing inflammation, oxidative damage, and protecting endothelium	NF-κB, MAPK	Li et al., 2019; Ma et al., 2019
	Endotoxic shock	in vivo	Anti-inflammatory	Inhibits NF-κB and NLRP3 inflammasome signaling pathways	NF-κB, NLRP3	Li et al. (2020a)
	Cerebral ischemia-reperfusion injury	in vitro, *in vivo*	Neuroprotective	Activates PPAR-γ, coordinating the nrf2/HO-1 antioxidant defense and suppressing NF-κB-driven neuroinflammation	PPAR-γ, nrf2/HO-1, NF-κB	Yao et al. (2022)
Fraxin	Organ protection and disease treatment					
	Radiation-induced intestinal injury	in vitro, *in vivo*	Intestinal protectant, promotes regeneration	Promotes IL-22 expression, protecting intestinal stem cells and barrier function	IL-22	Tang et al. (2024)
	Glucocorticoid-induced osteoporosis (GIOP)	in vitro, *in vivo*	Anti-osteoporotic, inhibits ferroptosis	Activates the Nrf2/GPX4 signaling axis, inhibiting osteoblast ferroptosis and promoting bone formation	Nrf2/GPX4	Zheng Z. et al. (2024)
	Liver injury	in vitro, *in vivo*	Hepatoprotective	Inhibits MAPK and NF-κB pathway activation, alleviating oxidative stress and inflammation	MAPK, NF-κB	Niu et al. (2017)
	Hyperuricemia	in vivo	Uricosuric	Acts as a ligand for the ABCG2 transporter, activating ABCG2 to promote uric acid excretion	ABCG2	Huang et al. (2023)
	Atherosclerosis	in vitro	Anti-atherosclerotic	Core target is TLR4; regulates the TLR4/PI3K/Akt pathway to inhibit oxidative stress and inflammation	TLR4/PI3K/Akt	Wang et al. (2025a)
	Skin whitening	in vitro, *in vivo*	Inhibits melanogenesis	Inhibits the ERK/MAPK pathway, downregulating melanogenesis-related proteins (MITF, TYR)	ERK/MAPK	Luo et al. (2023)
Fraxetin	Anti-tumor					
	Bladder cancer	in vitro, *in vivo*	Anti-proliferative, pro-apoptotic	Inhibits the Akt pathway, inducing apoptosis	Akt	Weng et al. (2024)
	Prostate cancer	in vitro	Anti-proliferative, anti-metastatic	Downregulates PLK4 expression, inhibiting the downstream PI3K/Akt signaling pathway	PLK4, PI3K/Akt	Ma et al. (2022)
Fraxetin	Organ protection					
	Cisplatin-induced nephrotoxicity	in vitro, *in vivo*	Nephroprotective	Activates FoxO1, alleviating DNA damage and inflammation	FoxO1	Yuan et al. (2025)
	Liver fibrosis	in vivo	Hepatoprotective, anti-fibrotic Inhibits tumor metabolism (warburg effect)	Modulates NF-κB/IκBα, MAPKs, and Bcl-2/Bax pathways, inhibiting inflammation and apoptosis	NF-κB/IκBα, MAPKs, Bcl-2/Bax	Wu et al. (2019)
Other metabolites	Other diseases					
Oleuropein	Hepatocellular carcinoma (HCC)	in vitro, *in vivo*	Inhibits tumor metabolism (warburg effect)	Directly binds to the key glycolytic enzyme GPI, inhibiting glycolysis	GPI	Hong, 2022; Wu, 2020
Total coumarins/Extract	Hyperuricemia	in vitro, *in vivo*	Uricosuric, metabolic regulation	Downregulates renal urate transporters URAT1/GLUT9; activates ABCG2; modulates gut microbiota	URAT1, GLUT9, ABCG2, gut microbiota	Zhou et al., 2018; Huang et al., 2023; Wang et al., 2016b; Liu and Liu, 2020
Total coumarins/Extract	Rheumatoid arthritis	in vivo	Anti-arthritic, anti-inflammatory	Reduces serum levels of TNF-α, IL-1β, and rheumatoid factor (RF)	TNF-α, IL-1β	Wu et al. (2024)
Total coumarins/Extract	Osteoporosis	in vivo	Anti-osteoporotic	Modulates the OPG/RANKL pathway, inhibiting osteoclast activity	OPG/RANKL	Liu M. J.et al. (2019)
Total coumarins/Extract	Hyperuricemia (metabolomics)	in vivo	Systemic metabolic regulator	Reverses multiple endogenous biomarker disturbances involved in amino acid, lipid, carbohydrate, and purine metabolism	Multi-target, holistic regulation	Wang et al. (2016)

VSMC: vascular smooth muscle cell; LDL: Low-Density Lipoprotein.

### 3.1 Anti-inflammatory and immunomodulatory effects

The “heat-clearing” efficacy of Fraxini Cortex in TCM theory is interpreted in modern science as potent anti-inflammatory activity, which underpins its therapeutic basis for dysentery, colitis, arthritis, and various inflammatory diseases. Its mechanisms are systematically elucidated, with its constituents achieving precise regulation of the inflammatory response by acting on upstream receptors, key kinases, and transcription factors within inflammatory signaling cascades.

First, isolated coumarins from Fraxini Cortex directly target the origin and hubs of inflammatory signaling pathways, including the TLR4 receptor and β2 integrin function. In a septic cardiomyopathy model, the purified compound esculin was confirmed through molecular docking and experimental validation to directly bind to Toll-like receptor 4 (TLR4), forming stable hydrogen bonds with amino acid residues such as VAL-308, ASN-307, CYS-280, CYS-304, and ASP-281. This binding inhibited LPS-induced TLR4 upregulation, subsequently blocking the phosphorylation of its downstream effector NF-κB p65, ultimately alleviating myocardial inflammation, oxidative stress, and apoptosis. The cardioprotective effect of esculin was abolished upon TLR4 plasmid overexpression, directly proving TLR4 as its key target (Su et al., 2024). In acute lung injury (ALI), the mechanism of esculin is particularly unique. Using drug affinity responsive target stability (DARTS) assay, *in vitro* protein binding experiments, and molecular docking, esculin was found to directly interact with β2 integrin on the neutrophil surface, thereby reducing its affinity for intercellular adhesion molecule-1 (ICAM-1). This led to suppressed Vav1 phosphorylation, hindered Rac1 activation, and subsequent inhibition of the PAK1/LIMK1/cofilin signaling axis, disrupting key processes required for cytoskeletal remodeling. This physically blocked neutrophil migration and chemotaxis, effectively alleviating ALI (Ni et al., 2023).

Concurrently, multiple metabolites of Fraxini Cortex synergistically inhibit core inflammatory pathways like NF-κB and MAPK. Esculin, fraxin, and esculetin have been confirmed in various inflammatory models (e.g., ulcerative colitis, ALI/ARDS, endotoxic shock, sepsis) to effectively inhibit NF-κB pathway activation, manifested as reduced IκBα degradation, suppressed p65 nuclear translocation, and a significant decrease in the production of downstream pro-inflammatory factors such as TNF-α, IL-6, and IL-1β (Su et al., 2024; Sun et al., 2024; Li et al., 2019; Li et al., 2020a; Li et al., 2016c; Tian et al., 2019). In ulcerative colitis and ALI models, fraxin also significantly reduced the phosphorylation levels of p38, JNK, and ERK in the MAPK pathway (Sun et al., 2024; Ma et al., 2019). In macrophage inflammation models, esculetin demonstrated multi-pathway synergistic anti-inflammatory capability by inhibiting not only NF-κB but also the activation of STAT1 and STAT3 (Cheng et al., 2021).

In a rat model of rheumatoid arthritis, the water decoction of Fraxini Cortex significantly reduced serum levels of TNF-α, IL-1β, and rheumatoid factor (RF), alleviating joint swelling and inflammatory cell infiltration, confirming its anti-arthritic effect (Wu et al., 2024). The anti-inflammatory activity of Fraxini Cortex extract has also been applied in daily products. Studies show that toothpaste containing Fraxini Cortex extract significantly inhibited xylene-induced ear swelling in mice, suggesting its potential in preventing and treating oral inflammation (Liao et al., 2022).

Furthermore, Fraxini Cortex metabolites inhibit NLRP3 inflammasome activation and pyroptosis. In endotoxic shock and ALI models, fraxin downregulated the expression of NLRP3 inflammasome-related proteins and inhibited caspase-1 activation and IL-1β maturation (Li et al., 2020a; Li et al., 2019). In myocardial ischemia-reperfusion injury (MIRI), esculin protected the myocardium by activating the Akt/GSK3β signaling pathway, which in turn inhibited NF-κB activation and NLRP3 inflammasome assembly, ultimately reducing GSDMD-mediated pyroptosis. The cardioprotective effect of esculin was reversed by the Akt inhibitor MK-2206, confirming the critical role of this pathway (Xu et al., 2021).

Fraxini Cortex metabolites also modulate anti-inflammatory nuclear receptors such as PPARγ. In a DSS-induced ulcerative colitis model, esculin promoted PPAR-γ nuclear localization while inhibiting NF-κB activity. PPARγ activation is recognized as an important anti-inflammatory mechanism, forming cross-regulation with the NF-κB pathway to jointly alleviate colonic inflammation (Tian et al., 2019).

Regarding immunomodulation, in an allergic asthma mouse model, esculetin alleviated airway inflammation and hyperresponsiveness by regulating the Th1/Th2 immune balance, reducing levels of IL-4 and immunoglobulin E (IgE) in bronchoalveolar lavage fluid while increasing interferon-γ (IFN-γ) levels, demonstrating anti-asthmatic potential (Li et al., 2020b).

Esculetin also induced apoptosis in rat vascular smooth muscle cells, a mechanism associated with downregulating Bcl-2 expression and upregulating Bax and caspase-9 expression, which may play a role in treating vascular inflammatory diseases (Li, 2015).

While numerous *in vitro* and *in vivo* studies confirm the inhibitory effects of Fraxini Cortex constituents on core inflammatory pathways like NF-κB and MAPK, important limitations should be noted. Most studies are cell-based, using compound concentrations (typically 10–100 μM) that may not be achievable *in vivo* given the poor oral bioavailability of coumarins. Furthermore, inconsistencies exist regarding the relative potency of esculin versus esculetin across different experimental models, which may reflect differences in cell types, stimulation protocols, or compound purity. Head-to-head comparative studies under standardized conditions are needed to clarify these discrepancies. Additionally, the translation of these findings to humans remains largely unexplored, representing a critical knowledge gap.

### 3.2 Antioxidant stress and ferroptosis

Fraxini Cortex metabolites play key roles in oxidative stress-related diseases and tumor therapy by activating endogenous antioxidant defense systems and precisely regulating ferroptosis. The purified coumarins esculetin and esculin are widely confirmed as potent activators of the Nrf2 pathway. They promote Nrf2 nuclear translocation and upregulate the expression of a series of downstream antioxidant enzymes, such as heme oxygenase-1 (HO-1), glutathione peroxidase 4 (GPX4), superoxide dismutase (SOD), and glutathione peroxidase (GSH-Px), thereby enhancing the cell’s ability to scavenge reactive oxygen species (ROS) and resist oxidative damage (Cai and Cai, 2023; Luo et al., 2023; Yang et al., 2025; Ju et al., 2024). In a glucocorticoid-induced osteoporosis (GIOP) model, the protective effect of fraxin on osteoblasts was confirmed to be achieved by activating the Nrf2/GPX4 signaling axis. Knocking down Nrf2 with siRNA completely blocked the protective effect of fraxin, directly proving the necessity of Nrf2 in this process (Zheng Z. et al., 2024).

Ferroptosis is a novel form of iron-dependent, lipid peroxidation-driven cell death. Fraxini Cortex metabolites exhibit remarkable bidirectional regulatory capabilities in different pathological contexts. In terms of anti-tumor effects, the mechanism of esculetin in hepatocellular carcinoma (HCC) involves inhibiting the Nrf2-xCT/GPX4 axis. Specifically, esculetin treatment led to decreased expression of Nrf2 and its downstream targets xCT (responsible for cystine uptake) and GPX4 (responsible for repairing lipid peroxidation), causing intracellular Fe^2+^ accumulation, GSH depletion, elevated levels of lipid ROS and MDA, ultimately triggering ferroptosis. Overexpression of Nrf2 reversed esculetin-induced ferroptosis (Qu et al., 2025). In colorectal cancer (CRC), esculin triggered ferroptosis by inducing endoplasmic reticulum stress. It activated the ER stress sensor PERK, which phosphorylated eIF2α. PERK activation, on one hand, regulated the eIF2α/CHOP axis to induce apoptosis, and on the other hand, regulated the Nrf2/HO-1 axis, leading to HO-1 overexpression, intracellular iron overload, and ultimately ferroptosis. Knocking down HO-1 significantly attenuated esculin-induced ferroptosis (Ji et al., 2024).

Conversely, Fraxini Cortex metabolites inhibit ferroptosis to protect tissues. In the GIOP model, fraxin inhibited osteoblast ferroptosis via the aforementioned activation of the Nrf2/GPX4 axis, promoting bone formation (Zheng Z. et al., 2024). The mechanism of esculetin in an intracerebral hemorrhage (ICH) model is more novel. Studies found that esculetin promoted NUDT1‐mediated m^7^G methylation of GPX4 mRNA. This post-transcriptional modification enhanced GPX4 mRNA stability, increased GPX4 protein levels, thereby inhibiting neuronal ferroptosis and improving neurological deficits. Silencing GPX4 abolished the inhibitory effect of NUDT1 overexpression on ferroptosis (Gong et al., 2025).

This apparent duality—promoting ferroptosis in cancer cells while inhibiting it in normal tissues—can be explained by several factors: (i) Differential cellular baseline states: Cancer cells typically operate under elevated oxidative stress with constitutively activated Nrf2 as a survival mechanism; disrupting this adaptation (e.g., by inhibiting Nrf2) is sufficient to trigger cell death. In contrast, normal cells under pathological stress (e.g., glucocorticoid-induced oxidative damage) may have insufficient Nrf2 activity, requiring activation to restore homeostasis. (ii) Dose-dependent effects: Low concentrations of coumarins may induce adaptive antioxidant responses (hormesis), while higher concentrations directly impair mitochondrial function and promote cell death. (iii) Microenvironmental influences: The acidic, hypoxic tumor microenvironment can fundamentally alter cellular responses to the same compound compared to normal tissue conditions. This bidirectional regulatory capacity exemplifies the potential of Fraxini Cortex constituents as “homeostasis restorers” rather than simple inhibitors or activators—a concept consistent with the TCM principle of treating different conditions with the same botanical drug.

### 3.3 Anti-tumor effects

The anti-tumor effects of Fraxini Cortex involve inducing various forms of cell death, inhibiting proliferation and migration, and uniquely targeting metabolic vulnerabilities of tumors. Esculetin induced S-phase cell cycle arrest in human hepatocellular carcinoma BEL-7402 cells, downregulated the Bcl-2/Bax ratio, leading to decreased mitochondrial membrane potential, activation of Caspase-9 and Caspase-3, ultimately initiating the mitochondrial apoptosis pathway (Ji and Wei, 2015). In triple-negative breast cancer (TNBC), esculetin induced cancer cell apoptosis by inhibiting the JAK2/STAT3 signaling pathway, thereby removing its anti-apoptotic effect, and activating the Caspase cascade (Caspase-9, -3, PARP cleavage). It showed a synergistic effect with the JAK2/STAT3 inhibitor WP1066 (Feng et al., 2025). There is also an endoplasmic reticulum stress pathway: as mentioned, esculin strongly induced apoptosis in colorectal cancer by inducing ER stress and the PERK/eIF2α/CHOP pathway (Ji et al., 2024). Studies also found that a series of esculetin derivatives synthesized via chemical derivatization of esculetin (e.g., compounds obtained by esterification with cinnamic acid) exhibited stronger *in vitro* anti-tumor activity than esculetin itself, more effectively inhibiting the proliferation of human HepG2 and HCT-116 cells (Zhang, 2016).

Targeting tumor metabolic reprogramming—inhibiting the Warburg effect—is a distinctive mechanism identified in studies on Fraxini Cortex constituents, particularly oleuropein and esculetin. Using transcriptomics, network pharmacology, and ultrafiltration mass spectrometry screening, the purified compound oleuropein was found to directly bind to the key glycolytic enzyme GPI (glucose-6-phosphate isomerase) with a binding constant (Kd) of 107 μM. Interfering with GPI expression or oleuropein treatment both inhibited glucose consumption and lactate production in HepG2 cells, downregulating the gene and protein expression of multiple key glycolytic enzymes such as PFKL, ALDOA, PGK1, ENO1, PKM2, and LDHA, thereby inhibiting the tumor Warburg effect (Hong, 2022; Wu, 2020). Further research found that scopolin from Fraxini Cortex interfered with the protein-protein interaction between fatty acid synthase (FAS) and GPI. MST and co-immunoprecipitation experiments confirmed that scopolin disrupted the binding of FAS to GPI by altering FAS conformation, providing a novel perspective on the anti-tumor mechanism (Wu et al., 2020).

Additionally, Fraxini Cortex metabolites inhibit proliferation and metastasis-related pathways. In prostate cancer DU145 cells, fraxetin downregulated the expression of PLK4 (Polo-like kinase 4), thereby inhibiting its downstream PI3K/Akt signaling pathway, leading to suppressed cell proliferation, migration, invasion, and induced apoptosis. Overexpression of PLK4 reversed these effects of fraxetin (Ma et al., 2022). Fraxetin also exerted anti-tumor effects in bladder cancer by inhibiting the Akt pathway (Weng et al., 2024).

### 3.4 Regulation of uric acid, glucose, and lipid metabolism

#### 3.4.1 Promoting uric acid excretion

A water extract of Fraxini Cortex (particularly from Shaanxi province) significantly downregulated the protein and mRNA expression levels of key renal urate reabsorption transporters URAT1 and GLUT9, thereby reducing uric acid reabsorption in renal tubules (Zhou et al., 2018). On the other hand, Fraxini Cortex metabolites activate intestinal and renal uric acid excretion: Using bioaffinity ultrafiltration mass spectrometry (BA-UF-MS), the purified compound fraxin was successfully screened from the plasma of rats administered Fraxini Cortex as a ligand for the ABCG2 transporter. Subsequent functional experiments confirmed that fraxin activates ABCG2 function. ABCG2, highly expressed in the kidneys and intestines, is responsible for actively secreting uric acid into urine or the intestinal lumen, serving as a crucial urate excretion pump (Huang et al., 2023). Metabolomics studies based on ^1^H-NMR and LC-MS revealed that Fraxini Cortex could reverse 26 endogenous biomarker changes in hyperuricemic rats. These biomarkers are involved in multiple pathways including amino acid metabolism, lipid metabolism, purine metabolism, and carbohydrate metabolism, uncovering that Fraxini Cortex treats hyperuricemia by multi-targeted, holistic correction of metabolic disorders (Wang et al., 2016b). The total coumarins of Fraxini Cortex, as the main active fraction for treating hyperuricemia, were confirmed to have diuretic and uricosuric effects, providing a pharmacodynamic basis for its application in this field (Liu and Liu, 2020).

Notably, as a core metabolite, Fraxini Cortex plays a key role in gout-treating formulas (e.g., Qinpi Tongfeng Fang and Qigui Tongfeng Pian), with its uric acid-lowering and anti-inflammatory activities jointly contributing to efficacy (Zhang et al., 2017; Fan et al., 2024). Furthermore, in the pre-pharmaceutical research of Compound Ximing Capsule (composed of Thlaspi arvense and Fraxini Cortex) for gout treatment, Fraxini Cortex is also an important constituent, and the establishment of its quality standard ensures the preparation’s efficacy (Yu, 2015). Moreover, development of a fermented traditional Chinese medicine compound containing Fraxini Cortex, by increasing levels of active metabolites like resveratrol and esculetin and modulating gut microbiota, showed good effects in preventing poultry gout (Zhang et al., 2025c).

#### 3.4.2 Improving insulin resistance

The mechanism by which the purified coumarin esculin improves obesity-induced insulin resistance is unique. It does not achieve this by reducing body weight, but by improving adipose tissue remodeling: including reducing adipocyte size, increasing adipocyte number, decreasing collagen deposition and inflammation (reducing TNF-α), and promoting angiogenesis (increasing VEGF-A). Simultaneously, it directly activates the IRS1/PI3K/AKT/GLUT4 signaling pathway in adipocytes, promoting GLUT4 translocation to the cell membrane, thereby enhancing glucose uptake and improving systemic insulin sensitivity (Yang et al., 2024).

#### 3.4.3 Regulating lipid metabolism

In an ApoE^−/−^ atherosclerosis model, esculin was found to improve blood lipid metabolism (reducing TC, TG, LDL-C, increasing HDL-C) and alleviate arterial plaque formation and local inflammation by inhibiting the DPP4/NF-κB signaling pathway (Zhang L. L. et al., 2025a). Esculetin, as summarized in reviews, exerts anti-atherosclerotic effects through multiple mechanisms including lowering blood lipids, inhibiting vascular smooth muscle cell (VSMC) proliferation, anti-LDL oxidation, and reducing adhesion factor and chemokine secretion (Wang Q. H. et al., 2022 ). Fraxin, predicted by network pharmacology and verified *in vitro*, has TLR4 as its core target for anti-atherosclerosis, regulating the TLR4/PI3K/Akt pathway to inhibit ROS generation and the expression of pro-inflammatory factors (IL-1β, IL-6, TNF-α) (Wang et al., 2025a). Regarding hepatic lipid metabolism, esculin significantly reduced free fatty acid (FFA)-induced levels of malondialdehyde (MDA), alanine aminotransferase (ALT), and aspartate aminotransferase (AST) in hepatocytes, increased reduced glutathione (GSH), decreased intracellular lipid droplet accumulation and triglyceride (TG) content, thereby ameliorating hepatocyte steatosis, possibly through inhibiting the PERK/eIF2A/ATF4 ER stress signaling pathway (Xu et al., 2024).

### 3.5 Organ and tissue protection

Under various pathological conditions, Fraxini Cortex and its active constituents demonstrate significant protective effects across multiple organ systems, mediated through the precise regulation of key signaling pathways (Zheng Z. et al., 2024; Liu et al., 2018; Liu M. J. et al., 2019). In the skeletal system, fraxin counters glucocorticoid-induced osteoporosis by activating the Nrf2/GPX4 axis to inhibit osteoblast ferroptosis, while esculetin preserves bone mass by downregulating serum IL-6 and the RANKL/OPG pathway to suppress osteoclast activity (Zheng Z. et al., 2024; Liu et al., 2018; Liu M. J. et al., 2019). Hepatoprotection is achieved via dual mechanisms: directly, through fraxin’s inhibition of MAPK/NF-κB pathways and fraxetin’s modulation of NF-κB/IκBα, MAPKs, and Bcl-2/Bax to alleviate oxidative stress, inflammation, and fibrosis (Niu et al., 2017; Wu et al., 2019); and indirectly, via gut microbial metabolism of coumarins (e.g., fraxin to fraxetin to 6,7,8-trihydroxycoumarin) which exert stronger activity by modulating the gut-liver axis, a process further exemplified by the enzymatic hydrolysis of esculin to esculetin (Zhang, 2024; Zhang et al., 2025b; Yang, 2020). Neuroprotection involves distinct strategies: esculetin inhibits ferroptosis post-intracerebral hemorrhage by enhancing GPX4 mRNA stability via NUDT1 (Gong et al., 2025), fraxin combats cerebral ischemia-reperfusion injury by activating PPAR-γ to coordinate Nrf2/HO-1 antioxidant defense and suppress NF-κB-driven neuroinflammation (Yao et al., 2022), and esculin shows promise in neurodegenerative models by activating stress-resistance pathways (Wang Q. H. et al., 2022), with esculetin also being a candidate for ALS therapy (Deng, 2016). For renal protection, fraxetin mitigates cisplatin-induced acute kidney injury by promoting FoxO1-mediated DNA repair (Yuan et al., 2025). Intestinal health is supported by fraxin’s promotion of IL-22-dependent stem cell proliferation after radiation injury (Tang et al., 2024), the anti-diarrheal effect of its ethanol extract (Tsai et al., 2004), and the efficacy of related compound formulations in treating inflammatory diarrhea (Yu et al., 2025; Xia et al., 2017). Pulmonary protection is afforded by esculin and fraxin through inhibition of neutrophil migration and the NF-κB/MAPK/NLRP3 pathways, respectively, in acute lung injury models (Ni et al., 2023; Li et al., 2019; Ma et al., 2019). Finally, ocular protection is demonstrated by esculetin’s activation of the HIF-1α/BNIP3 pathway to induce protective autophagy in retinal pigment epithelial cells under high glucose stress (Chen et al., 2022). This multi-organ protective profile, unified by anti-inflammatory, antioxidant, and cytoprotective signaling, underscores the systemic therapeutic potential of Fraxini Cortex.

### 3.6 Other effects

Additionally, Fraxini Cortex and its active metabolites exhibit efficacy in skin whitening, antiviral, antibacterial, and insecticidal activities. Fraxin reduced melanin synthesis by inhibiting the ERK/MAPK pathway to downregulate melanogenesis-related proteins like MITF and TYR, while activating the NRF2 pathway to upregulate CAT and HO-1, scavenging H_2_O_2_-induced ROS and protecting melanocytes (Luo et al., 2023). Antivirally, fraxetin effectively inhibited dengue virus and Zika virus replication, a mechanism related to binding the viral envelope E protein and inhibiting viral adsorption (Liao, 2022); Fraxini Cortex water extract and esculetin also showed dose-dependent inhibitory effects on grouper iridovirus (Huang et al., 2021). Furthermore, using an ultrafiltration mass spectrometry combined with molecular docking screening strategy, metabolites like esculin from Fraxini Cortex were identified as potential inhibitors of influenza virus neuraminidase (reference 41 repeated, but no information provided). Coumarin metabolites from Fraxini Cortex (fraxetin, esculetin, esculin, etc.) exhibited significant killing activity against pine wood nematode (Bursaphelenchus xylophilus), a mechanism closely related to inhibiting acetylcholinesterase and Ca^2+^-ATPase activities, interfering with nerve conduction and muscle function (Feng, 2023).

### 3.7 Integrating the evidence: toward an immune-metabolic network perspective

**FIGURE 2:**
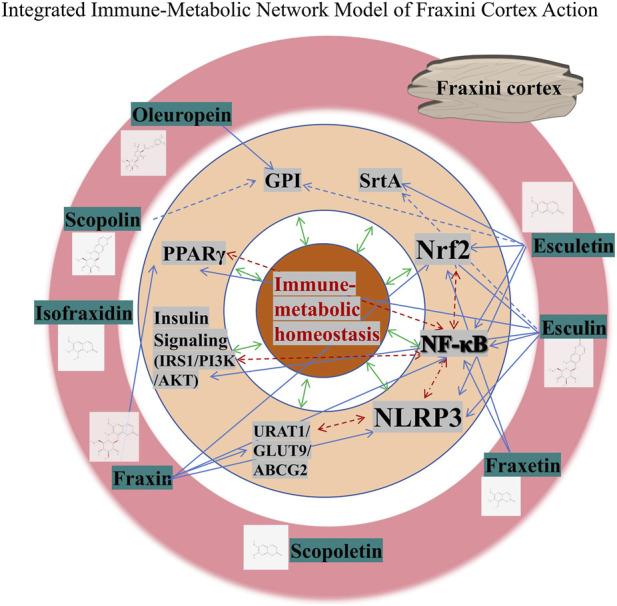
Integrated Immune-Metabolic Network Model of Fraxini Cortex Action. This schematic illustrates the proposed multi-target network through which constituents of Fraxini Cortex (Qinpi) converge to restore immune-metabolic homeostasis. The model is organized into three concentric layers: the outer layer lists major bioactive metabolites; the middle layer represents key molecular targets that interface between immune and metabolic pathways; and the innermost core signifies the ultimate functional outcome—immune-metabolic homeostasis. Colored arrows denote distinct types of interactions: Blue arrows: Direct or indirect connections from bioactive constituents to their corresponding molecular targets, based on experimental evidence. Green arrows: Connections from each target to the central core, indicating that modulation of these targets collectively contributes to the restoration of immune-metabolic homeostasis. Red bidirectional dashed arrows: Crosstalk interactions among the core targets, highlighting the interconnected nature of immune and metabolic signaling pathways.

Legend: This conceptual framework illustrates the proposed mechanism by which Qinpi acts as a multi-target regulator of immune-metabolic homeostasis. Key bioactive metabolites (left panel) interact with specific molecular targets in immune and metabolic pathways (central panels). These interactions converge to suppress pathological inflammation, correct metabolic dysfunction, and resolve oxidative stress, thereby promoting tissue repair and homeostasis. The resulting integrated effects (right panel) underlie its potential in treating diverse chronic diseases characterized by immune-metabolic dysregulation. Arrows indicate activation, inhibition, or modulation. Key representative references are indicated in parentheses. Abbreviations: GPI, glucose-6-phosphate isomerase; SrtA, Sortase A; TLR4, Toll-like receptor 4; NF-κB, nuclear factor kappa B; Nrf2, nuclear factor erythroid 2-related factor 2; URAT1, urate transporter 1; ABCG2, ATP-binding cassette subfamily G member 2.

The pharmacological actions of the multiple active metabolites of Fraxini Cortex suggest a new paradigm beyond the traditional single-target model. We propose a hypothesis: the core mechanism of action of Fraxini Cortex may lie in its ability to act as a “regulator of the immune-metabolic network.” This hypothesis is based on the convergence of the following observations (Figure 2): (i) its metabolites can directly target key nodes connecting metabolism and immunity, such as the glycolytic enzyme GPI and the bacterial virulence factor synthase SrtA; (ii) it effectively corrects systemic metabolic disorders like hyperuricemia and insulin resistance, which themselves are drivers of chronic inflammation; (iii) all these effects ultimately converge on the inhibition of classic inflammatory signaling pathways like NF-κB and NLRP3. For example, the inhibition of urate reabsorption via URAT1/GLUT9 not only corrects hyperuricemia but also reduces the formation of monosodium urate crystals, a potent trigger of inflammation (NLRP3 activation). Similarly, targeting GPI in cancer cells cuts off their energy supply (a metabolic effect) and may also disrupt the extracellular cytokine-like functions of GPI that promote inflammation and angiogenesis. Although these lines of evidence still require more direct experimental validation under the unified model of “immune-metabolic crosstalk,” this framework provides an insightful new perspective for understanding Fraxini Cortex’s potential in treating multiple diseases with the same therapy and for designing combination therapies for complex chronic diseases.

## 4 Preliminary clinical translation and evidence

Despite the compelling and multifaceted pharmacological mechanisms revealed by extensive preclinical studies, a significant translational gap exists between these findings and established clinical application for Fraxini Cortex. A systematic screening of current evidence identifies only a limited number of clinical studies meeting rigorous inclusion criteria, underscoring the urgent need for high-quality human trials (Jiang et al., 2025; Zhao et al., 2015; Chen and Zhao, 2022; Tan, 2018; Lu et al., 2022; Fan et al., 2022) (Table 3). Nonetheless, these preliminary investigations offer valuable, hypothesis-generating insights into its therapeutic potential across several disease domains, which intriguingly align with its proposed role as an immune-metabolic regulator.

**TABLE 3:** Summary of clinical evidence for cortex fraxini and its preparations.

Medical condition/Focus of study	Intervention/Bioactive compound	Study design	Population and sample size (I/C)	Key primary outcomes and findings [instrument/Metric]	Evidence level and critical remarks	REF
Plaque psoriasis (psoriasis vulgaris)	Fraxini cortex (Qin pi) medicinal bath (as an adjunct to NB-UVB therapy)	Randomized, controlled trial	60 patients (30/30)	1. Significantly higher efficacy rate in the combination group (96.67%) vs. NB-UVB alone group (70.00%) 2. Treatment response was defined as lesion area improvement >30%	Level II Remarks: Demonstrates the significant adjunctive benefit of fraxini cortex bath with standard phototherapy. Suggests a synergistic effect for skin lesions. The specific contribution of its bioactive compounds warrants further investigation	Zhao et al. (2015)
Dry eye disease	Jiawei Qinpi decoction (compound formula containing fraxini cortex)	Randomized, single-blind, controlled trial	90 patients (45/45) 180 eyes (90/90)	1. Significantly greater improvement in OSDI, FBUT, SIT, FL scores, and meibomian gland function vs. saline control 2. Higher total efficacy rate (84.44% vs. 62.22%) 3. Significant reduction in HADS scores for anxiety and depression	Level II Remarks: Demonstrates efficacy for a specific TCM syndrome. Suggests benefits for both ocular surface parameters and mood. The contribution of individual botanical drug metabolites cannot be delineated	Jiang et al. (2025)
Asthenopia (eye strain)	Esculin and digitalisglycosides eye drops (standardized preparation)	Randomized, controlled trial	80 patients (40/40)	1. Significantly higher efficacy rate vs. lifestyle advice control (92.5% vs. 62.5%) 2. Greater reduction in subjective asthenopia score 3. Significant improvement in objective accommodation function: Amplitude, lag, and facility	Level II Remarks: Demonstrates efficacy for asthenopia by improving ciliary muscle function and accommodation. Good safety profile reported. Provides clinical evidence for a modern ophthalmic preparation	Chen and Zhao (2022)
Computer vision syndrome (Video terminal syndrome)	Esculin and digitalisglycosides eye drops (standardized preparation) plus fusion training	Prospective, controlled cohort study	86 patients (43/43)	1. Combination therapy significantly reduced multiple symptom scores (dryness, itch, pain, blur, etc.) vs. fusion training alone 2. Higher overall efficacy rate (76.74% vs. 55.81%) 3. Improved patient comfort score, tear film stability (BUT), and secretion (schirmer test)	Level III Remarks: Highlights the additive benefit of the phytochemical drops to visual training. Improves both functional symptoms and objective tear film parameters. Long-term efficacy and safety require further study	Tan (2018)
Acute gouty arthritis (AGA)	Qinpi tongfeng formula (QPTFF) (A multi-herbal formula with cortex fraxini as the monarch botanical drug) plus Bloodletting therapy	Randomized, Assessor-Blinded, controlled trial (study protocol)	86 patients (planned)	Primary outcome: Total effective rate. The study is designed as a non-inferiority trial against colchicine. Aims to demonstrate synergistic effects of internal herbal medicine and external therapy	Level I (protocol) Remarks: High-quality study protocol. Aims to provide evidence for a TCM combination therapy. The specific contribution of cortex fraxini is supported by its role as the monarch botanical drug and its known anti-inflammatory and uric acid-lowering pharmacology	Lu et al. (2022)
Acute gouty arthritis (AGA)	Qinpi tongfeng formula (QPTFF) (A multi-herbal formula with cortex fraxini as the monarch botanical drug)	Double-blind, double-dummy, multicenter, randomized, non-inferiority controlled trial	114 patients (57/57) 105 completed (53/52)	1. Analgesic effect: Non-inferior to diclofenac sodium (VAS score change; 95% CI lower limit > −0.7) 2. Uric acid Reduction: Significantly greater reduction in serum uric acid vs. control 3. Safety: Significantly lower treatment-related adverse events (TRAEs: 7.02% vs. 26.32%)	Level I Remarks: Provides high-quality evidence that QPTFF is an effective and safer alternative to NSAIDs for AGA, with the added benefit of uric acid reduction. The multi-herbal composition targets multiple pathways	Fan et al. (2022)

Emerging clinical data, though limited, suggest beneficial effects of Fraxini Cortex preparations: (1) Inflammatory Skin Disease: As an adjunct to narrow-band UVB therapy, a Fraxini Cortex medicinal bath significantly enhanced the clinical response rate in patients with plaque psoriasis (96.67% vs. 70.00% with UVB alone, P = 0.006) (Zhao et al., 2015). (2) Ocular Surface Disorders: A modified Qinpi decoction improved signs and symptoms of dry eye disease, including OSDI scores, tear film break-up time, and Schirmer test values, while also alleviating comorbid anxiety and depression (Jiang et al., 2025; Chen and Zhao, 2022). Furthermore, a standardized eye drop containing its active metabolite esculin demonstrated efficacy in relieving asthenopia and computer vision syndrome by improving accommodative function (Tan, 2018). (3) Acute Gouty Arthritis (AGA): The Qinpi Tongfeng Formula (QPTFF), with Fraxini Cortex as the principal botanical drug, demonstrated non-inferior analgesic effects to diclofenac sodium in patients with AGA. Notably, QPTFF showed a superior uric acid-lowering effect and a significantly lower incidence of treatment-related adverse events (7.02% vs. 26.32%, P < 0.05), highlighting a potentially favorable benefit-risk profile that integrates anti-inflammatory and urate-lowering actions (Fan et al., 2022; Lu et al., 2022).

As summarized in Table 3, the existing clinical evidence is predominantly exploratory. Most studies are small-sample, single-center trials with limited description of randomization methods and allocation concealment, introducing potential bias. Only the QPTFF trial for acute gouty arthritis (Fan et al., 2022) employed a rigorous double-blind, double-dummy, multicenter design, lending greater credibility to its findings. Notably, the disease targets implicated—psoriasis (immune dysregulation), dry eye (inflammatory metabolite), and gout (a canonical immunometabolic disorder)—are precisely the conditions where an agent capable of modulating both inflammatory and metabolic pathways would be predicted to be effective. The efficacy of QPTFF in gout is particularly resonant, as it mirrors the dual anti-inflammatory (e.g., NF-κB inhibition) and metabolic-corrective (URAT1/GLUT9/ABCG2 modulation) mechanisms elucidated in preclinical models.

**FIGURE 3:**
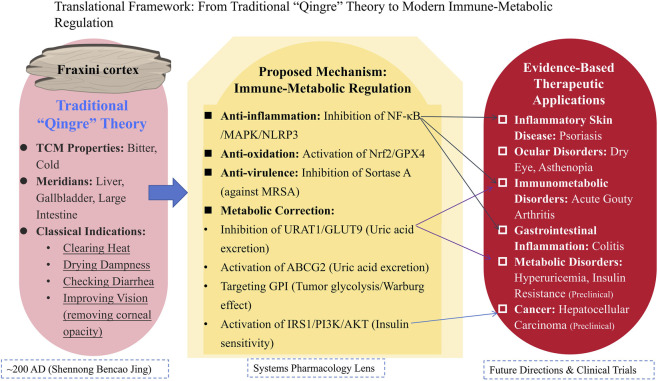
Translational Framework: From Traditional “Qingre” Theory to Modern Immune-Metabolic Regulation. This figure maps traditional TCM indications of Fraxini Cortex (left) to modern disease categories (right) through the unifying lens of immune-metabolic regulation. The central pathway illustrates the molecular mechanisms that bridge traditional concepts with contemporary understanding, providing a roadmap for evidence-based clinical translation.

The stark contrast between the rich,multi-target pharmacopoeia of Fraxini Cortex and the paucity of robust clinical evidence defines the primary challenge for its future development (Figure 3). The existing low-level clinical evidence serves not as confirmation, but as a crucial justification for investing in targeted, mechanism-informed clinical trials. This gap directly informs the research priorities outlined in the following discussion section.

## 5 Pharmacokinetics and safety

### 5.1 Pharmacokinetic (ADME) profile

#### 5.1.1 Absorption and distribution

Studies indicate that its major coumarin metabolites (e.g., esculin, fraxin, esculetin, fraxetin) are rapidly absorbed after oral administration, with time to peak concentration (Tmax) mostly within 0.5–1 h (Zhao et al., 2016; Wang Y. et al., 2017). However, the plasma concentrations of the parent compounds are generally low, suggesting significant first-pass effect or intestinal metabolism (Zhao, 2015; Li C. X. et al., 2022).

Data on tissue distribution remain limited, representing a knowledge gap.

#### 5.1.2 Metabolism

Extensive metabolism *in vivo* is a core feature of Fraxini Cortex’s pharmacokinetics. Its metabolic pathways are diverse: Phase I metabolism primarily includes hydrolysis (e.g., conversion of esculin to esculetin by intestinal flora or esterases (Zhang, 2024; Luo, 2016), hydroxylation, and dehydroxylation; Phase II metabolism mainly involves glucuronidation and sulfation conjugation reactions, serving as the primary elimination route for parent compounds (Wang, 2017; Zhao, 2015). An in-depth study using HPLC-QTOF-MS/MS technology revealed that up to 52 metabolites could be identified in rats administered Fraxini Cortex water extract, far exceeding the 28 parent metabolites, fully illustrating the complex biotransformation network these metabolites undergo *in vivo* (Wang, 2017).

#### 5.1.3 Excretion

This extensive metabolism directly affects its excretion process, with metabolites primarily excreted renally via urine. In rat models, most coumarins and their metabolites are essentially completely excreted within 32 h post-dose, but the renal excretion rate of parent drugs is typically low (<10%). Esculetin is an exception, with a parent excretion rate reaching 19.26%, likely related to the substantial *in vivo* conversion of its precursor, esculin (Zhao, 2015). Biliary excretion is only a minor pathway (Zhao, 2015).

#### 5.1.4 Effect of pathological states

Notably, the body’s pathological state significantly influences the pharmacokinetic behavior of Fraxini Cortex. Comparative pharmacokinetic studies showed significant differences in the pharmacokinetic parameters (e.g., AUC, Cmax, CL) of major Fraxini Cortex metabolites between hyperuricemic model rats and normal rats, possibly due to renal impairment or altered drug-metabolizing enzyme activity under pathological conditions (Wang Y. et al., 2017). This finding suggests that the clinical use of Fraxini Cortex must fully consider the patient’s pathophysiological state (Wang Y. et al., 2017).

### 5.2 Formulation technology and strategies to enhance bioavailability

Addressing the issue of low oral bioavailability of Fraxini Cortex metabolites, researchers have explored effective enhancement strategies across multiple levels, from raw material processing to dosage form design.

In the extraction and purification stage, various novel, green, and efficient techniques have been successfully applied. For example, technologies such as ultrasonic-assisted deep eutectic solvent extraction (Wang Y. et al., 2020), ultrasonic-assisted supramolecular solvent microextraction (Ye et al., 2024), ionic liquid-synergistic ultrasonic-microwave extraction (Wang F. et al., 2017), and microwave-assisted enzymatic extraction (Li et al., 2016b) can significantly improve the extraction yield of target coumarin metabolites from Fraxini Cortex. Additionally, macroporous adsorption resin (e.g., ADS-5 type) purification technology has proven effective in enriching total coumarins from Fraxini Cortex, increasing their purity and providing high-quality raw materials for subsequent formulation development (Zhang et al., 2024; Wang et al., 2015b). Regarding processing techniques, integrated origin-processing and preparation technology, compared to traditional segmented processing, better preserves active ingredients like esculin and reduces loss, embodying the concept that “good medicinal materials are the foundation of good medicine” (Zhao et al., 2018a; Zhao et al., 2018b; Wang J. Y. et al., 2017).

In the construction of novel drug delivery systems, hydrogel technology shows great potential. Studies have successfully constructed esculin-loaded sodium carboxymethyl cellulose/carboxymethyl chitosan hydrogels. This system possesses a three-dimensional network structure that significantly retards drug release, prolongs the duration of action, and exhibits excellent antibacterial activity and biocompatibility, making it particularly suitable for treating local diseases like endometritis (Ding, 2024; Ding et al., 2024b; Ding et al., 2024a). In the field of ocular drug delivery, a developed Fraxini Cortex thermosensitive *in situ* forming gel can gelify at physiological eye temperature, significantly prolonging drug residence time in the eye, thereby improving bioavailability and providing a superior dosage form choice for treating ocular diseases like dry eye syndrome (Dun et al., 2016).

### 5.3 Safety assessment and research gaps

Fraxini Cortex demonstrates a favorable safety profile in both traditional use and modern toxicological research. Traditional Chinese medicine follows the principle of “discontinuing when the disease is resolved,” with a common clinical decoction dose range of 6–30 g, within which safety is good (Zhao et al., 2024; Zhu et al., 2021). Its main contraindication is for patients with a TCM syndrome pattern of “deficiency-cold of the spleen and stomach” to prevent damage from its bitter-cold nature, which may cause gastrointestinal discomfort (Zhao et al., 2024).

Modern toxicological studies provide strong support for its safety. At the organ level, specialized safety pharmacology evaluations showed no significant toxicity of esculetin to the cardiovascular, respiratory, or central nervous systems at effective doses (Luo, 2016). At the animal model level, effective metabolites of Fraxini Cortex, such as esculetin and fraxin, not only showed significant anti-fibrotic and hepatoprotective effects in CCl_4_-induced liver fibrosis and injury models but also caused no observable toxic damage to liver and kidney functions or major organs (Wu et al., 2019; Niu et al., 2017). Furthermore, the active metabolite oleuropein from Fraxini Cortex exhibited significant anti-hepatocellular carcinoma effects in tumor-bearing mouse models without observed hepatorenal toxicity (Hong, 2022).

However, despite this generally favorable profile, several critical research gaps must be addressed: (i) Long-term toxicity data: Although existing studies support safety in acute and sub-acute settings, systematic long-term toxicity studies (e.g., chronic carcinogenicity, reproductive toxicity) for whole Fraxini Cortex extract and its major constituents are lacking. (ii) Drug interaction potential: As coumarin-rich compounds, Fraxini Cortex constituents may theoretically affect cytochrome P450 enzymes (e.g., CYP1A2, CYP3A4) or drug transporters (e.g., P-gp). While no specific interaction reports exist, caution is warranted when co-administering with drugs having narrow therapeutic windows (e.g., warfarin). The confirmed synergistic antibacterial effect between esculetin and vancomycin represents a beneficial interaction, but systematic drug interaction studies are needed. (iii) Special population considerations: Given that coumarins undergo extensive hepatic metabolism, dose adjustment studies in patients with hepatic impairment are lacking. Similarly, safety in pregnancy and lactation has not been systematically evaluated. (iv) Coumarin-related concerns: While Fraxini Cortex coumarins differ structurally from the hepatotoxic coumarins found in some other plants (e.g., Melilotus), systematic evaluation of coumarin-specific toxicity remains warranted.

## 6 Discussion and future perspectives

When positioned alongside other representative heat-clearing botanical drugs, Fraxini Cortex exhibits a distinctive immune-metabolic duality. For example, Coptis chinensis (Huanglian, containing berberine) demonstrates anti-inflammatory and lipid-lowering effects, whereas its modulation of uric acid transporters (URAT1/ABCG2) remains largely unreported—an area where Fraxini Cortex has shown potential. Scutellaria baicalensis (Huangqin, containing baicalin) is renowned for anti-inflammatory and antiviral activities, but the anti-virulence strategy targeting SrtA against MRSA and the metabolic targeting of GPI in cancer have been predominantly reported in constituents of Fraxini Cortex, with no parallel evidence currently identified in Scutellaria baicalensis or other commonly studied heat-clearing botanical drugs. This distinctive target profile positions Fraxini Cortex as particularly suited for conditions at the intersection of inflammation and metabolic dysregulation, such as gout, metabolic syndrome-associated inflammation, and certain cancers.

A substantial body of evidence supports the reconceptualization of Fraxini Cortex from a broad-spectrum “heat-clearing” agent to a precise immunometabolic modulator. We propose that this paradigm is supported by several converging lines of evidence. Firstly, its direct targeting of metabolic enzymes with immune consequences, such as GPI in tumor glycolysis (Wu et al., 2020; Hong, 2022) and SrtA in bacterial virulence (Li et al., 2020a), demonstrates its capacity to disrupt pathological processes at their metabolic roots. Secondly, its regulation of metabolite transporters like URAT1, GLUT9, and ABCG2 (Huang et al., 2023; Zhou et al., 2018; Wang et al., 2025b) directly corrects metabolic imbalances—such as hyperuricemia—that are both a cause and consequence of inflammation. Thirdly, its systemic amelioration of insulin resistance by improving adipose tissue remodeling and activating the IRS1/PI3K/AKT/GLUT4 pathway (Yang et al., 2024) shows a restorative effect on core metabolic homeostasis, which in turn suppresses chronic low-grade inflammation. These actions are unified by fundamental anti-inflammatory and antioxidant mechanisms (e.g., NF-κB/MAPK inhibition, Nrf2 activation) that protect tissues from collateral damage during immunometabolic dysregulation (Cai and Cai, 2023; Ju et al., 2024; Hu et al., 2024). Consequently, the efficacy of Fraxini Cortex in models of arthritis, colitis, metabolic disease, and cancer can be reinterpreted not as disparate effects, but as manifestations of its core competency—recalibrating the immunometabolic network—within specific pathological contexts.

Adopting the “immunometabolic modulator” framework opens unique and innovative avenues for future research, moving beyond incremental studies. Future work must employ single-cell multi-omics (transcriptomics, metabolomics) to decipher how Fraxini Cortex constituents differentially affect specific immune cell subsets (e.g., M1 vs. M2 macrophages, Th17 vs. Treg cells) and their metabolic states (e.g., glycolysis, oxidative phosphorylation) within diseased tissues like arthritic joints or the tumor microenvironment. Spatial transcriptomics could map these effects *in situ*. Genetic validation using CRISPR-Cas9 to knockout or activate predicted key targets (e.g., GPI, SrtA, Nrf2) in relevant cell types is crucial for establishing causality within the complex phytochemical milieu (Feng et al., 2025; Qu et al., 2025).

The most promising and unique preclinical findings should then inform clinical trial design. For instance: (1) Anti-virulence Strategy: Conduct exploratory clinical trials to assess whether Fraxini Cortex extract (or esculetin) as an adjuvant therapy can improve outcomes and reduce antibiotic use in patients with methicillin-resistant *Staphylococcus aureus* (MRSA) skin or soft tissue infections, leveraging its SrtA inhibition and immunomodulatory effects (Li et al., 2020a). (2) Metabolism-Targeted Therapy: Based on its mechanism of inhibiting the Warburg effect via GPI targeting (Hong, 2022), investigate the potential of active Fraxini Cortex metabolites combined with chemotherapy or immune checkpoint inhibitors for treating GPI-high tumors (e.g., hepatocellular carcinoma), focusing on tumor microenvironment reprogramming and enhanced efficacy. (3) Homeostasis-Restoring Therapy: For immunometabolic disorders like ulcerative colitis, conduct multicenter RCTs using standardized, bioavailability-optimized Fraxini Cortex preparations to evaluate their efficacy in the maintenance of remission. Endpoints should include endoscopic healing, changes in specific metabolite profiles, and gut microbiota composition.

Concurrently, the development of next-generation derivatives and smart formulations is imperative. Lead compounds like esculin and esculetin serve as excellent starting points for medicinal chemistry optimization. Structure-activity relationship (SAR) studies should focus on improving pharmacokinetic properties (e.g., metabolic stability, membrane permeability) while maintaining or enhancing affinity for key targets like GPI or SrtA. This could yield first-in-class small-molecule inhibitors of bacterial virulence or tumor metabolism derived from a natural product. Advanced drug delivery systems are also critical. Beyond traditional formulations, research should develop stimuli-responsive nanocarriers (e.g., ROS-sensitive, pH-sensitive) that can selectively release active constituents at inflammatory or tumor sites, thereby maximizing local efficacy and minimizing systemic exposure.

Finally, quality control must evolve from merely quantifying marker compounds to incorporating bioactivity-based assays that reflect the proposed immunometabolic modulatory function. For example, standardizing extracts based on their *in vitro* potency to inhibit LPS-induced NF-κB activation in macrophages or modulate GPI activity would ensure pharmacological consistency between batches. Furthermore, sustainable cultivation practices (Good Agricultural Practice) and ecological studies are needed to ensure the long-term availability of high-quality raw materials, as genotype, geography, and processing methods influence chemical composition and bioactivity (Zhou, 2021; Zhao et al., 2018a).

We acknowledge several limitations in the current evidence base and this review. First, our proposed “immune-metabolic regulator” framework, while supported by convergent evidence, remains a hypothesis requiring direct experimental validation in unified systems (e.g., simultaneous assessment of immune and metabolic parameters in the same disease model). Second, the predominance of *in vitro* and animal studies limits direct translational inference; physiologically relevant concentrations and human data are urgently needed. Third, the complexity of multi-metabolite interactions remains incompletely characterized—synergistic, additive, or antagonistic effects among constituents warrant systematic investigation using modern network pharmacology approaches.

In conclusion, Fraxini Cortex stands at a crossroads between its rich history and a promising future defined by modern systems biology. By redefining its essence as a systemic immunometabolic modulator, we offer a cohesive scientific narrative that explains its myriad effects, validates traditional wisdom in contemporary mechanistic terms, and, most importantly, charts a clear and innovative path forward for research. This paradigm shift is essential for unlocking the full potential of Fraxini Cortex and transforming it from a traditional botanical drug into a well-characterized, evidence-based medicine for modern, complex, and interconnected diseases.
