# Supplementary material for: Fraxini cortex (Qinpi): reframing a traditional heat-clearing botanical drug as a systemic immune-metabolic regulator
Source: Front Pharmacol. 2026 Jun 17;17:1799355. doi: 10.3389/fphar.2026.1799355 (PMC13318886; doi:10.3389/fphar.2026.1799355)
Supplement: Supplementary file 1 [file Supplementaryfile1.docx]

**Supplementary Material：**

**Literature Search Strategy and Study Selection Criteria**

1. Literature Search Strategy

A comprehensive literature search was conducted from inception until January 1, 2026, across the following electronic databases: PubMed, Web of Science (Core Collection), Embase, Cochrane Central Register of Controlled Trials (CENTRAL), China National Knowledge Infrastructure (CNKI), Wanfang Data, and China Biology Medicine disc (CBM). The search strategy was designed to combine Medical Subject Headings (MeSH) or equivalent controlled vocabulary terms with free-text words in titles and abstracts. No language restrictions were applied initially, but only studies published in Chinese or English were considered for inclusion during the screening phase.

Example search strategy for PubMed:

("Fraxinus"[Mesh] OR "Fraxinus"[tiab] OR "Fraxini Cortex"[tiab] OR "Ash Bark"[tiab] OR "Qin Pi"[tiab] OR "Qinpi"[tiab] OR "Cortex Fraxini"[tiab]) OR ("Esculin"[Mesh] OR esculin[tiab] OR esculoside[tiab] OR "aesculin"[tiab]) OR ("Esculetin"[Mesh] OR esculetin[tiab] OR "aesculetin"[tiab] OR "cichorigenin"[tiab] OR "6,7-dihydroxycoumarin"[tiab]) OR ("Fraxetin"[Supplementary Concept] OR fraxetin[tiab] OR "7,8-dihydroxy-6-methoxycoumarin"[tiab]) OR ("Fraxin"[tiab] OR "fraxin"[tiab]) OR ("oleuropein"[tiab] OR "secoiridoid*"[tiab])) AND (("Immunomodulation"[Mesh] OR immunomodulat*[tiab] OR "immune regulat*"[tiab] OR "anti-inflammatory"[tiab] OR antiinflammatory[tiab] OR "Immunity"[Mesh] OR immun*[tiab]) OR ("Metabolism"[Mesh] OR metaboli*[tiab] OR "metabolic regulat*"[tiab]) OR ("Gout"[Mesh] OR gout[tiab] OR "hyperuricemia"[tiab] OR "uric acid"[tiab] OR "URAT1"[tiab] OR "GLUT9"[tiab] OR "ABCG2"[tiab]) OR ("Diabetes Mellitus"[Mesh] OR "Insulin Resistance"[Mesh] OR "diabetes"[tiab] OR "insulin resistance"[tiab] OR "glucose metabolism"[tiab] OR "lipid metabolism"[tiab] OR "GLUT4"[tiab]) OR ("Neoplasms"[Mesh] OR "anti-tumor"[tiab] OR antitumor[tiab] OR anticancer[tiab] OR "neoplasm*"[tiab] OR "GPI"[tiab] OR "glucose-6-phosphate isomerase"[tiab] OR "Warburg effect"[tiab]) OR ("Osteoporosis"[Mesh] OR osteoporosis[tiab] OR "bone loss"[tiab]) OR ("Dermatitis"[Mesh] OR "Psoriasis"[Mesh] OR "skin disease*"[tiab] OR psoriasis[tiab]) OR ("NF-kappa B"[Mesh] OR "NF-κB"[tiab] OR "nuclear factor kappa B"[tiab]) OR ("MAP Kinase Signaling System"[Mesh] OR "MAPK"[tiab] OR "p38 MAPK"[tiab] OR "JNK"[tiab] OR "ERK"[tiab]) OR ("NLR Family, Pyrin Domain-Containing 3 Protein"[Mesh] OR "NLRP3"[tiab] OR "inflammasome"[tiab]) OR ("NF-E2-Related Factor 2"[Mesh] OR "Nrf2"[tiab] OR "NFE2L2"[tiab]) OR ("Oxidative Stress"[Mesh] OR "oxidative stress"[tiab] OR "antioxidant*"[tiab]) OR ("Ferroptosis"[tiab] OR "iron-dependent cell death"[tiab]))

Example search strategy for CNKI (Chinese search formula example):

(SU='秦皮' OR TI='秦皮' OR KY='秦皮' OR AB='秦皮' OR SU='白蜡树皮' OR TI='白蜡树皮' OR AB='白蜡树皮' OR SU='Fraxini Cortex' OR AB='Fraxini Cortex' OR TI='Cortex Fraxini' OR AB='Cortex Fraxini') OR (SU='七叶苷' OR TI='七叶苷' OR AB='七叶苷' OR SU='七叶灵' OR AB='七叶灵' OR TI='Esculin' OR AB='Esculin' OR SU='秦皮甲素' OR AB='秦皮甲素') OR (SU='七叶内酯' OR TI='七叶内酯' OR AB='七叶内酯' OR SU='秦皮乙素' OR AB='秦皮乙素' OR TI='Esculetin' OR AB='Esculetin' OR SU='6,7-二羟基香豆素' OR AB='6,7-二羟基香豆素') OR (SU='白蜡树精' OR TI='白蜡树精' OR AB='白蜡树精' OR TI='Fraxetin' OR AB='Fraxetin' OR SU='秦皮素' OR AB='秦皮素') OR (SU='白蜡苷' OR TI='白蜡苷' OR AB='白蜡苷' OR TI='Fraxin' OR AB='Fraxin') OR (TI='橄榄苦苷' OR AB='橄榄苦苷' OR TI='Oleuropein' OR AB='Oleuropein')) AND ((SU='免疫调节' OR TI='免疫调节' OR AB='免疫调节' OR SU='免疫' OR TI='免疫' OR AB='免疫' OR SU='抗炎' OR TI='抗炎' OR AB='抗炎') OR (SU='代谢' OR TI='代谢' OR AB='代谢' OR SU='代谢调节' OR AB='代谢调节') OR (SU='痛风' OR TI='痛风' OR AB='痛风' OR SU='高尿酸血症' OR TI='高尿酸血症' OR AB='高尿酸血症' OR SU='尿酸' OR AB='尿酸' OR TI='URAT1' OR AB='URAT1' OR TI='GLUT9' OR AB='GLUT9' OR TI='ABCG2' OR AB='ABCG2') OR (SU='糖尿病' OR TI='糖尿病' OR AB='糖尿病' OR SU='胰岛素抵抗' OR TI='胰岛素抵抗' OR AB='胰岛素抵抗' OR SU='糖代谢' OR AB='糖代谢' OR SU='脂代谢' OR AB='脂代谢' OR TI='GLUT4' OR AB='GLUT4') OR (SU='肿瘤' OR TI='肿瘤' OR AB='肿瘤' OR SU='抗癌' OR TI='抗癌' OR AB='抗癌' OR SU='抗肿瘤' OR AB='抗肿瘤' OR TI='GPI' OR AB='GPI' OR TI='葡萄糖-6-磷酸异构酶' OR AB='葡萄糖-6-磷酸异构酶' OR AB='瓦博格效应') OR (SU='骨质疏松' OR TI='骨质疏松' OR AB='骨质疏松' OR SU='骨丢失' OR AB='骨丢失') OR (SU='皮肤病' OR TI='皮肤病' OR AB='皮肤病' OR SU='银屑病' OR TI='银屑病' OR AB='银屑病') OR (SU='NF-κB' OR TI='NF-κB' OR AB='NF-κB' OR SU='核因子κB' OR AB='核因子κB') OR (SU='MAPK' OR TI='MAPK' OR AB='MAPK' OR SU='丝裂原活化蛋白激酶' OR AB='丝裂原活化蛋白激酶') OR (SU='NLRP3' OR TI='NLRP3' OR AB='NLRP3' OR SU='炎症小体' OR AB='炎症小体') OR (SU='Nrf2' OR TI='Nrf2' OR AB='Nrf2') OR (SU='氧化应激' OR TI='氧化应激' OR AB='氧化应激' OR SU='抗氧化' OR AB='抗氧化') OR (SU='铁死亡' OR TI='铁死亡' OR AB='铁死亡'))

2. Literature Screening and Synthesis Approach

Literature screening was conducted independently by two investigators. Initially, titles and abstracts were screened to exclude studies clearly irrelevant to the theme of Fraxinus Cortex (Qinpi) and immune-metabolic regulation (e.g., studies focusing on other herbs or non-related diseases). Subsequently, the full texts of potentially eligible articles were reviewed for final judgment based on the pre-defined inclusion and exclusion criteria. For evidence synthesis, we prioritized studies that provided clear mechanistic insights into the immunomodulatory and/or metabolic regulatory effects of Fraxinus Cortex and its constituents. Data pertaining to its phytochemistry, multi-target pharmacological actions (e.g., on GPI, SrtA, URAT1/GLUT9/ABCG2, NF-κB/Nrf2 pathways), pharmacokinetics, and clinical outcomes were extracted and synthesized to construct the core narrative of this review.

3. Study Selection Criteria

Inclusion Criteria:

(i) Original research studies (in vitro, animal, or human clinical investigations) examining Fraxinus Cortex (Qinpi) and/or its major bioactive constituents (e.g., esculin, esculetin, fraxin, fraxetin, oleuropein);

(ii) Studies focusing on its immunomodulatory, anti-inflammatory, metabolic regulatory (e.g., anti-gout, anti-diabetic, anti-cancer), or organ-protective effects;

(iii) Studies that directly investigated or discussed mechanisms related to the proposed paradigm of immune-metabolic regulation.

Exclusion Criteria:

(i) Studies unrelated to Fraxinus Cortex, its constituents, or the core themes of immunology and metabolism;

(ii) Conference abstracts, commentaries, editorials, and other non-original research publications;

(iii) Duplicate publications.
